# Estimating PM_2.5_ utilizing multiple linear regression and ANN techniques

**DOI:** 10.1038/s41598-023-49717-7

**Published:** 2023-12-19

**Authors:** Sumita Gulati, Anshul Bansal, Ashok Pal, Nitin Mittal, Abhishek Sharma, Fikreselam Gared

**Affiliations:** 1 Department of Mathematics, S. A. Jain College, Ambala, Haryana 134003 India; 2 Department of Chemistry, S. A. Jain College, Ambala, Haryana 134003 India; 3 Department of Mathematics, Chandigarh University, Gharuan, Mohali, 140413 India; 4 University Centre for Research and Development, Chandigarh University, Gharuan, Mohali, 140413 India; 5 Department of Computer Engineering and Applications, GLA University, Mathura, 281406 India; 6 Faculty of Electrical and Computer Engineering, Bahir Dar Institue of Technology, Bahir Dar University, Bahir Dar, Ethiopia

**Keywords:** Environmental sciences, Engineering

## Abstract

The accurate prediction of air pollutants, particularly Particulate Matter (PM), is critical to support effective and persuasive air quality management. Numerous variables influence the prediction of PM, and it's crucial to combine the most relevant input variables to ensure the most dependable predictions. This study aims to address this issue by utilizing correlation coefficients to select the most pertinent input and output variables for an air pollution model. In this work, PM_2.5_ concentration is estimated by employing concentrations of sulfur dioxide, nitrogen dioxide, and PM_10_ found in the air through the application of Artificial Neural Networks (ANNs). The proposed approach involves the comparison of three ANN models: one trained with the Levenberg–Marquardt algorithm (LM-ANN), another with the Bayesian Regularization algorithm (BR-ANN), and a third with the Scaled Conjugate Gradient algorithm (SCG-ANN). The findings revealed that the LM-ANN model outperforms the other two models and even surpasses the Multiple Linear Regression method. The LM-ANN model yields a higher R^2^ value of 0.8164 and a lower RMSE value of 9.5223.

## Introduction

Air is an admirable and most valued resource. It is the essential source on this earth that supports all living beings to survive and sustain. Unfortunately, in current years, due to several human exercises our valuable natural resources are getting contaminated. Air pollution is the most substantial environmental concern in almost all parts of the world. Due to the extensive advancements in economy around the globe, air quality evolves into a major issue as the diminishing air quality has incessant and somber effects not only on human health but also on the ecosystem. The WHO stated that around 90% of the world's population is inhaling polluted air (www.who.in). Moreover, the State of the Global Air report (2019), addresses air pollution as the fifth dominant hazard for mortality across the globe. Main pollutants that affect most of the nation comprises of particulate matter (PM), nitrogen dioxide (NO_2_), lead (Pb), carbon monoxide (CO), ozone (O_3_), sulphur dioxide (SO_2_) etc.. Raising levels of these deadly pollutants due to industrial activities, vehicles, construction sites, power plants, and natural processes like volcanoes, forest fires, has considerable brunt on human wellbeing. The deteriorated quality of air results in several types of aversions, cardiovascular diseases, respiratory ill health, etc.^1–3^. Owing to the numerous adverse effects on human well-being, this environmental issue must be considered significantly. Moreover, now a days many nations are collaborating to address the issue of increasing air pollution^4^.

The primitive pollutant affecting human health is PM. PM is composed up primarily of smoke, dust and soot or liquid droplets discharged into the environment from industries, vehicles, construction spots etc. The particles having an aerodynamic diameter lower than 2.5 μm are fine, PM_2.5_ and those having a diameter less than 10 μm are known as coarse particles, PM_10_. The pollutant deeply penetrates the respiratory system and the blood streams leading to many health hazards^5,6^. Hence, it is imperative to develop effective tools for monitoring PM levels, disseminating information regarding hazardous concentrations, and providing recommendations for preventive measures to mitigate such levels. Numerous investigations have been carried out, encompassing not only the quantification of PM levels but also the evaluation of potential health hazards associated with heightened PM exposure for the population. These studies greatly enhance our understanding of the complex public health challenges caused by the widespread effects of air pollution^7–16^.

Further, investigating the entire involved parameters contributing air pollution is an arduous task. To deal with this, air pollution models are desired to evolve early warnings and command actions and further to examine forthcoming ensuing discharge schemes^17,18^. The increasing role of machine learning in air quality prediction represents a significant leap forward in our ability to monitor and manage environmental health. Machine learning techniques have ushered in a new era of air quality forecasting, allowing us to harness vast amounts of data, including historical air quality information, meteorological data, and even satellite imagery. These algorithms can identify complex patterns and correlations within this data, enabling more accurate predictions of air quality parameters such as PM concentrations, ozone levels, and pollutant concentrations. By providing real-time, high-resolution forecasts, machine learning models empower policymakers, environmental agencies, and the public to make informed decisions, take preventive measures, and mitigate the adverse effects of air pollution on public health and the environment. The growing integration of machine learning into air quality prediction signifies a promising avenue for advancing our understanding of air pollution dynamics and enhancing the quality of life for communities around the world. Among various statistical procedures, Artificial Neural Networks (ANNs) have been demonstrated to be altogether effective for appropriating complex relationships and enhancing forecast accuracy^19–24^.

ANNs are computational models inspired by the structure and function of the human brain. They consist of interconnected nodes, or artificial neurons, organized into layers. These networks are used for various machine learning tasks, including pattern recognition, classification, regression, and even more complex tasks like natural language processing and image recognition. In ANNs, information flows through the network, with each neuron processing and transmitting data to the next layer. ANNs have gained widespread popularity due to their ability to handle complex and high-dimensional data, making them a crucial component of modern artificial intelligence and deep learning applications. They have been instrumental in advancing fields such as computer vision, speech recognition, and autonomous systems, among many others. Broadly, different kinds of ANN involve the back-propagation neural network^25,26^, multilayer perceptron^27,28^, radial basis function^29,30^, and adaptive neuro-fuzzy inference systems^31,32^.

The primary objective of this study is to assess the performance of ANN trained with different algorithms for predicting PM_2.5_ concentration. Additionally, we have conducted a comparative analysis with the traditional multiple linear regression model (MLR).

## Background

Numerous researchers have engaged in the thorough evaluation of air quality prediction models, with a specific emphasis on the precision of PM concentration predictions across a wide spectrum of scenarios, employing ANN. The use of ANNs for estimating PM concentration has been asserted for the prediction of hourly and daily average concentrations relying on air pollutants and atmospheric data^33,34^. In the Santiago city of Chile, Perez et al.^35^ demonstrated estimations of hourly average concentrations of PM_2.5_ several hours before, depending on values attained at a steady site. Further, outcomes acquired employing ANN revealed estimated errors within the extent 30–60%. Moreover, they examined the noise cutback of dataset to enhance predictions as imperative. A comparison of ANN technique with classical regression techniques for PM_10_ and PM_2.5_ estimation was conducted by McKendry^36^. He established that meteorological variables, endurance, and co-pollutant values effectively estimated PM levels. In another investigation, Chelani et al.^37^ entrenched an ANN procedure to predict PM_10_ and noxious metals contamination investigated in the Jaipur city of India. Authors were adept at estimating contaminations quite justly. Tecer^38^ suggested ANNs to estimate PM levels in Zonguldak Province, Turkey. The outcomes revealed that the suggested technique can effectively be employed to estimate air quality. Pires et al.^39^ demonstrated the accomplishment of five linear models to estimate the daily average PM_10_ levels and certified that the size of the dataset is an imperative factor for the estimation of models. Paschalidou et al.^40^ employed multilayer perceptron for PM_10_ hourly levels prediction in Cyprus. The prediction revealed that the MLP models displays the best estimation performance. Also, Roy et al.^41^ have suggested the utilization of both multiple regression and ANN techniques for analyzing PM levels in different seasons at a vast opencast coal mine in India. The findings indicated that the ANN-based forecasting outperformed the multiple regression models. An online air pollutants predicting ANN technique that utilizes parameters attained through geographic modeling for the district Besiktas, Instanbul was suggested by Kurt & Oktay^42^. This system employs the meteorological parameters, the air pollutants levels and certain area specific attributes as input parameters. The ANN technique was carried out in this study to develop PM_2.5_ concentration prediction model. In Spain, another ANN model for PM_10_ daily levels estimation was suggested that executes the estimation of a 24 h average PM_10_ levels and employs deterministic variables for overall transit of aerosols from arid areas^43^. An innovative approach was employed to forecast PM_2.5_ and PM_10_ levels in major Chinese cities. This approach integrated a feedforward ANN model with a rolling criterion to capture input data patterns and a cumulative generating conduct of gray model to reduce data sample unpredictability^44^. The prediction procedure relied mainly on the daily values of PM_2.5_ and PM_10_ levels and on a few atmospheric parameters. With an aim to analyze the impact of exposure to PM_10_ on health and to estimate PM_10_ levels using ANN another study was conducted in Yasuj city^45^. The daily average values of PM_10_ as well as the climatic data was utilized in this analysis. In general, amongst all the machine learning approaches, ANN has been proven to be the most favorable approach of the researchers. This study examined ANN technique with varying training functions to establish the most effective model for PM_2.5_ estimation.

## Methodology

### Study area and air quality data

**Figure 1:**
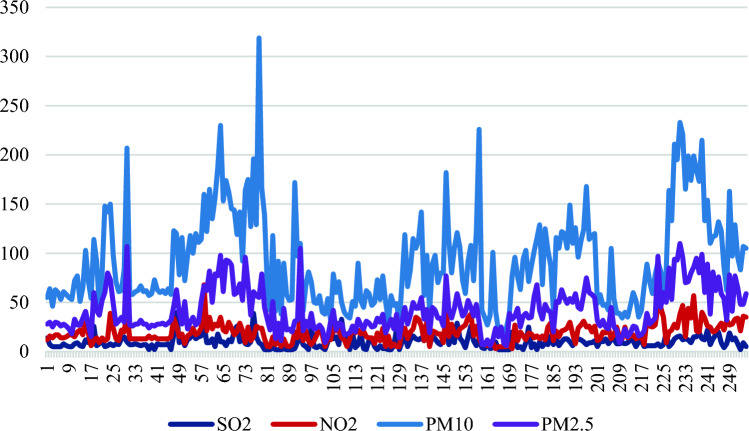
Values of air pollutant variables for the year 2021.

In India, central pollution control board (CPCB) is the pinnacle institution that investigates and monitors air quality. This institution supervises air pollution with the support of its abundant stations extended in nearly every city. Air quality across the country is systematically monitored through a combination of Manual and Continuous Ambient Air Quality monitoring stations. At present, this network comprises a total of 1257 monitoring stations. Manual monitoring activities are undertaken at 883 stations, encompassing 378 cities and towns distributed across 28 States and 7 Union Territories. Simultaneously, continuous monitoring is carried out at 374 stations, situated in 190 cities and towns across 27 States and 4 Union Territories. To facilitate the monitoring of air pollutants, the responsibility is shared with various entities such as the State Pollution Control Boards (SPCB), Pollution Control Committees (PCC), and other reputable institutions. The CPCB collaborates with these organizations to ensure the uniformity and consistency of air quality data while offering technical and financial support. It identifies and calculates pollutants as well as atmospheric parameters. Moreover, the monitoring of air pollutants is enforced with the support of SPCB, PCC, and several other reputed organizations. CPCB work with these assisting institutes to provide uniform, consistent air quality data^46^. The data generated through manual and continuous monitoring integrated for the year 2021 has been taken for this study involving the annual average values of SO_2_, NO_2_, PM_10_ and PM_2.5_ (in μg/m^3^) as shown in Fig. 1.

### Modeling and opting suitable input variables

**Figure 2:**
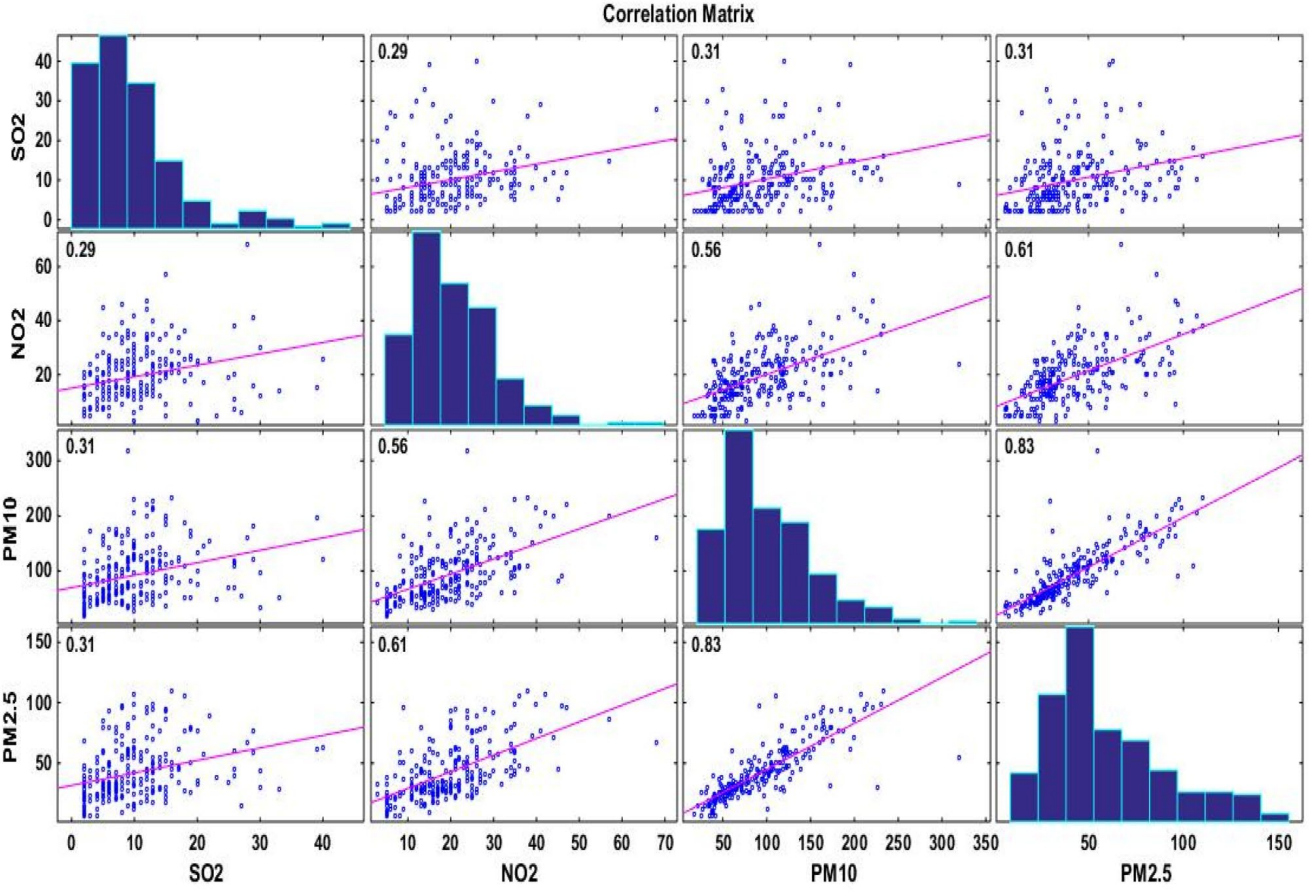
Correlation matrix of air pollutants in India for the year 2021.

The observed levels of air pollutants PM_10_, PM_2.5_, NO_2_, and SO_2_ were investigated with an objective to frame an air pollution estimation model. The specific dataset was sourced from CPCB for the year 2021. Figure 2 visually represents the relationships among SO_2_, NO_2_, PM_10_, and PM_2.5_ levels. We observed a positive correlation among all these variables, signifying their relevance to the study. Notably, the maximum correlation values were found to be 0.31 for PM_2.5_ with SO_2_, 0.61 with NO_2_, and 0.83 with PM_10_. Consequently, SO_2_, NO_2_, and PM_10_ were selected as the input variables for the PM_2.5_ air pollution estimation model.

### Estimating PM_2.5_

#### Multiple linear regression (MLR) model

Multiple linear regression (MLR) is a statistical technique used to assess the relationships between a single dependent variable and two or more independent variables. The method works by fitting a linear equation to the data, with coefficients representing the contribution of each independent variable to the dependent variable. The model aims to find the best-fitting line through the data points, which minimizes the sum of the squared differences between the observed and predicted values. MLR is a valuable tool for uncovering complex associations and understanding the underlying factors that influence a particular phenomenon. The formula for expressing the output dependent variable y in terms of independent variables x_1_, x_2_, …, x_n_ is as follows:

1 $$y={\alpha }_{0}+{\alpha }_{1}{x}_{1}+{\alpha }_{2}{x}_{2}+\dots +{\alpha }_{n}{x}_{n}+\varepsilon ,$$

where n = number of observations, ${\alpha }_{0}$ = the y intercept, ${\alpha }_{n}$= coefficient of the independent variable ${x}_{n}$ and $\varepsilon$= model error.

In this particular investigation, PM_2.5_ is taken as a dependent variable, while SO_2_, NO_2_ and PM_10_ are considered as independent variables. The MLR model computes the coefficients ${\alpha }_{1},$ ${\alpha }_{2}$,…,${\alpha }_{n}$ using the least square method.

#### Proposed ANN models

**Figure 3:**
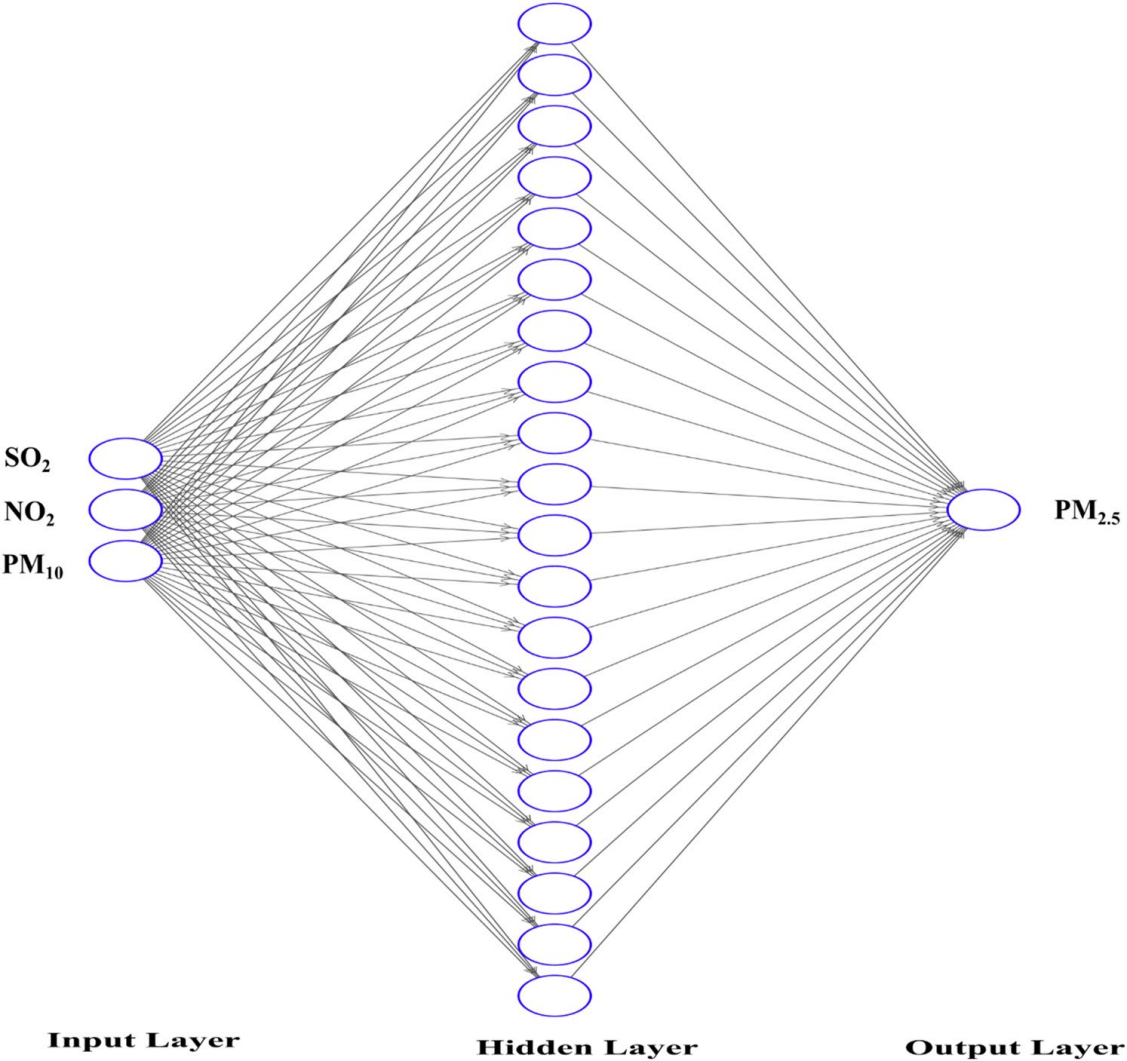
The structure of the ANN layers.

In the present analysis, the nftool of MATLAB (Version R2014b) was used and executed on a system equipped with an Intel HD Graphics card, a 17-inch display, 4 GB of memory, an Intel 11th generation i5 430 M processor, and a 512 GB SSD^47–49^. The ANN were trained using NO_2_, SO_2_, and PM_10_ as input variables and PM_2.5_ as the target variable. The neural network architecture consisted of 20 neurons in the hidden layer, as depicted in Fig. 3. For the initial training phase, the dataset was partitioned into training (70%), validation (10%), and testing (20%) subsets. ANNs employ various training algorithms to adjust the network's parameters (weights and biases) in order to minimize errors and improve performance. In this study, the network underwent training using three distinct algorithms sequentially: Levenberg–Marquardt, Bayesian Regularization, and Scalar Conjugate training algorithms.

##### Performance metrics

The assessment and differentiation of the MLR and the three proposed Artificial Neural Network (ANN) models are carried out by examining the Root Mean Square Error (RMSE) and Coefficient of Determination (R^2^). These metrics are defined as follows:

$$\mathrm{RMSE }= \sqrt{\frac{1}{n}} \sum_{i=1}^{n}{(o\left(t\right)-p\left(t\right))}^{2},$$

$${{\text{R}}}^{2} =1-\frac{\sum_{i=1}^{n}{\left(o\left(t\right)-p\left(t\right)\right)}^{2}}{\sum_{i=1}^{n}{\left(o\left(t\right)-\frac{1}{n}\sum_{i=1}^{n}o\left(t\right)\right)}^{2}},$$

where, n = number of observations, o(t) = actual value of the variable , p(t) = predicted value of the variable.

## Simulation results and discussion

This study focuses on the estimation of PM_2.5_ concentrations using annual average data of SO_2_, NO_2_, and PM_10_ for the year 2021 as input parameters. The performance evaluation of the models was based on the Root Mean Square Error (RMSE) and the coefficient of determination (R^2^). These metrics provided insights into the effectiveness of the MLR model and the three ANN models: ANN trained using the Levenberg–Marquardt algorithm (LM-ANN), the Bayesian Regularization algorithm (BR-ANN), and the Scaled Conjugate Gradient algorithm (SCG-ANN).

The experiments entail a comparison between the MLR model and three ANN models. The results of this comparison, specifically the evaluation metrics RMSE and R^2^, are presented in Table 1 for reference.

**Table 1:** Statistical error indices.

Model	RMSE	R^2^
LM-ANN	**9.5223**	**0.8164**
BR-ANN	9.6555	0.8118
SCG-ANN	11.0165	0.7551
MLR	11.7585	0.7201

Bold values indicate the best value from others.

The results revealed that the LM-ANN model outperformed the others, yielding the lowest RMSE of 9.5223 compared to 9.6555, 11.0165, and 11.7585 for BR-ANN, SCG-ANN, and MLR, respectively. Furthermore, the PM_2.5_ concentration estimated by the LM-ANN model demonstrates a strong correlation with observed values, with an R^2^ of 0.8164. In contrast, BR-ANN exhibits an R^2^ value of 0.8118, while SCG-ANN yields 0.7551, and MLR results in 0.7201.

**Figure 4:**
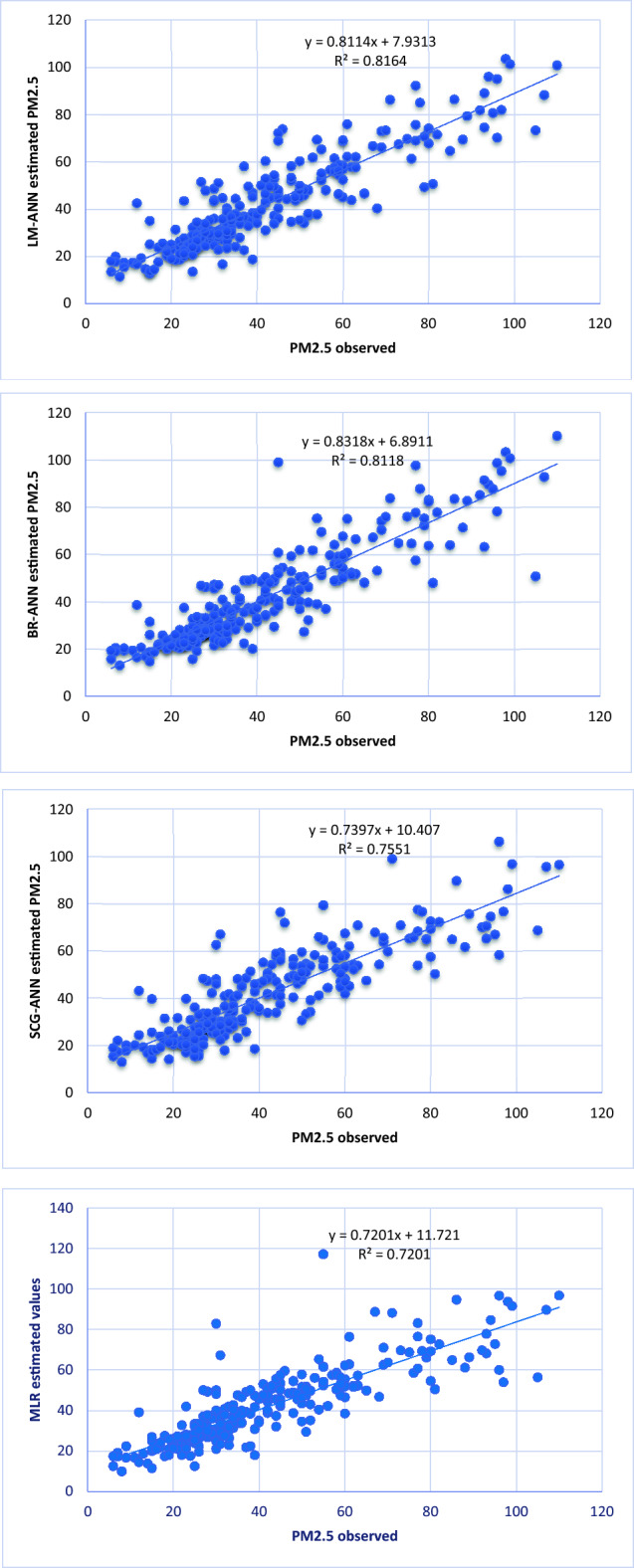
Correlation amongst observed and estimated PM_2.5_.

**Figure 5:**
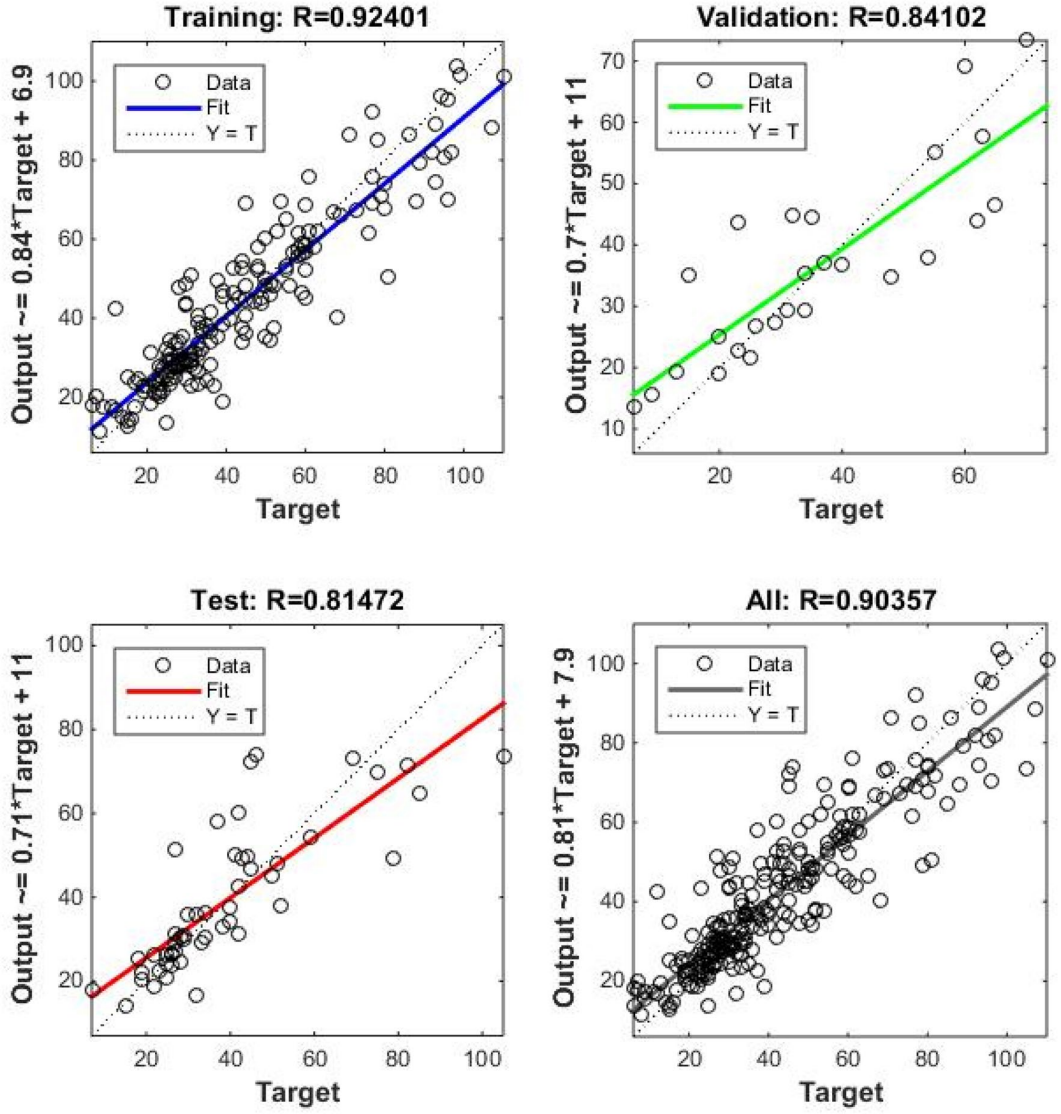
Regression plot of LM-ANN.

**Figure 6:**
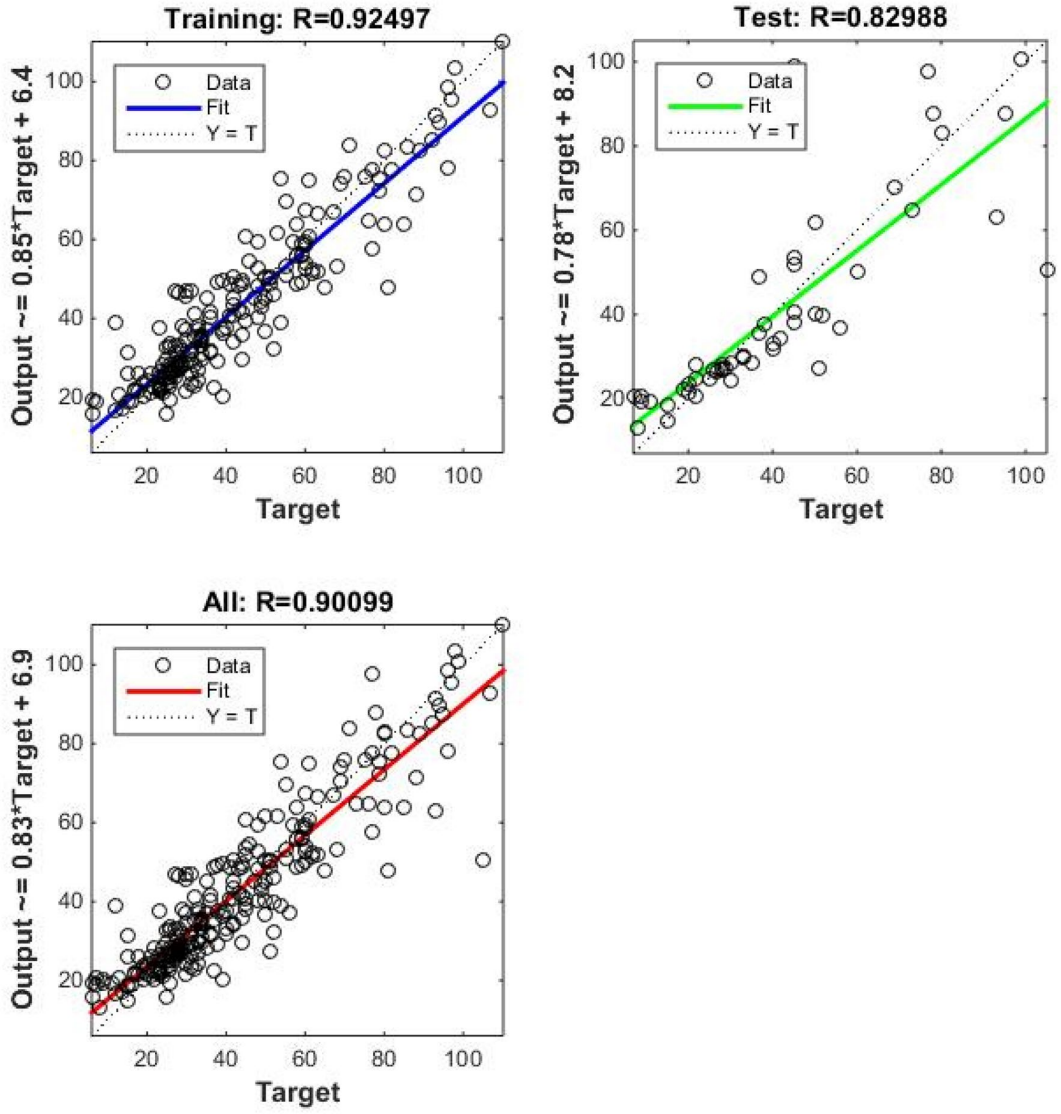
Regression plot of BR-ANN.

**Figure 7:**
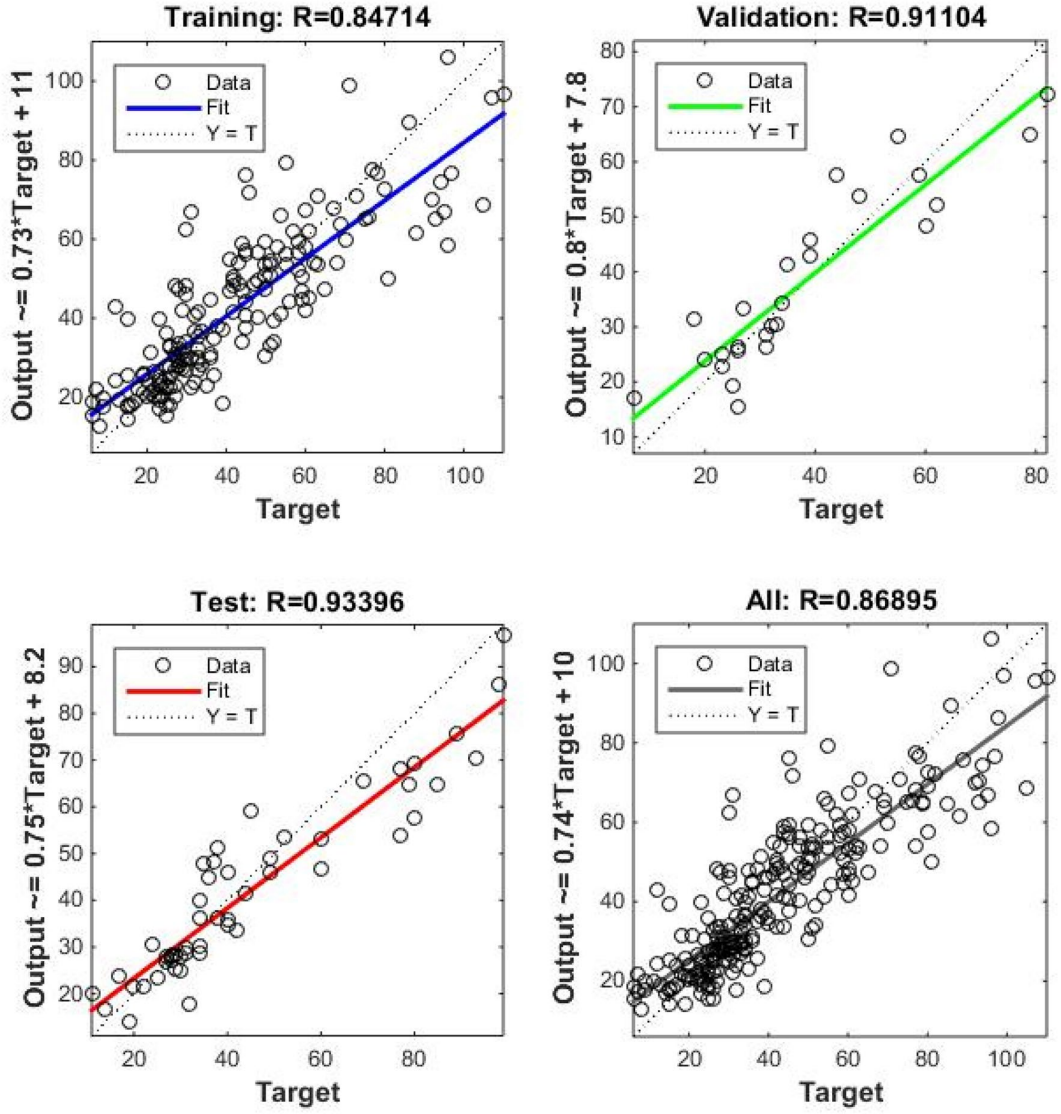
Regression plot of SCG-ANN.

**Figure 8:**
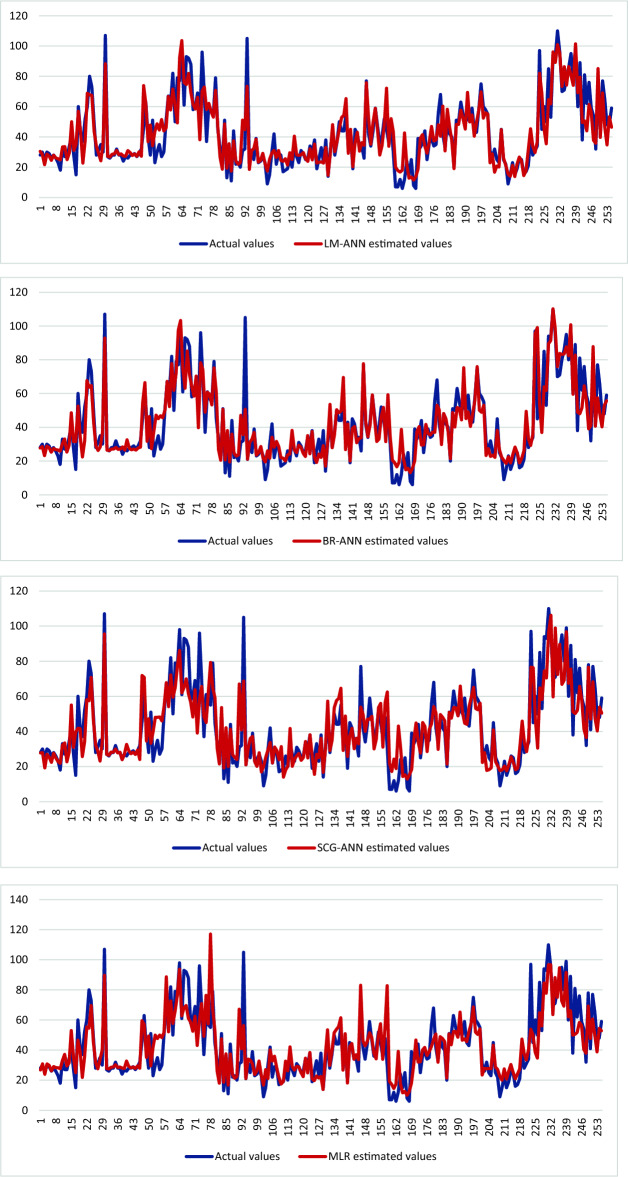
Comparison of the observed and estimated values of PM_2.5_.

Correlation amongst observed and estimated LM-ANN, BR-ANN SCG-ANN and MLR models are illustrated in Fig. 4. Additionally, Figs. 5, 6, and 7 represent the regression plots for the LM-ANN, BR-ANN, and SCG-ANN models, respectively. Moreover, Fig. 8 provides a time series illustration of the detected and estimated PM_2.5_ values for the suggested models.

Notably, the results highlighted the superior performance of the LM-ANN model in comparison to the other models, signifying its enhanced capability in estimating PM_2.5_ concentrations.

The current investigation offers a comprehensive exploration of the effectiveness of various ANN techniques when applied to the air quality modeling. This research not only sheds light on the adequacy of different ANN methodologies but also delves into their relative strengths and weaknesses in the context of air quality modeling. Consistency and size of data used, alongwith upholding of identical controlling factors for training and testing data are some of the limitations of proposed ANN models. By examining these diverse ANN approaches, we gain a deeper understanding of how they perform and contribute to the field of air quality modeling.

## Conclusion and future scope

Particulate matter (PM) is a major air pollutant known to have detrimental impacts on human health. This study involved the predictive analysis of PM_2.5_ levels by utilizing Artificial Neural Network (ANN) models based on data concerning SO_2_, NO_2_, and PM_10_ concentrations. The three distinct ANN models, namely LM-ANN, BR-ANN, and SCG-ANN were applied to India's air quality dataset for the year 2021 sourced from the CPCB. The error metrics, specifically Root Mean Square Error (RMSE) and R-squared (R^2^) were employed to assess the performance of these models. The findings demonstrated that the LM-ANN model exhibited superior performance compared to the other two ANN models and the Multiple Linear Regression (MLR) model. Moreover, these models have the potential to alert the public when PM concentration surpasses its prescribed level. Furthermore, the suggested models can be deployed to forecast real-time air quality trends using historical data, making them valuable tools for proactive planning and management of air pollution concerns. In summary, this ANN modeling approach offers a practical solution for governmental agencies to address air pollution issues and formulate effective strategies for mitigating their impact.

Regarding future research directions, we aim to extend our investigations into air pollution by integrating daily and hourly data, thereby enabling a more exhaustive analysis of pollution levels across diverse urban areas. Additionally, doe to the prominent performance exhibited by the LM-ANN model, there is potential for further enhancements to fine-tune its capabilities in air quality prediction.

## Data Availability

The data set utilized for this study is available on https://cpcb.nic.in/.

## References

[1] Kampa M, Castanas E (2008). Human health effects of air pollution. Environ. Pollut..

[2] Qin G, Meng Z (2009). Effects of sulfur dioxide derivatives on expression of oncogenes and tumor suppressor genes in human bronchial epithelial cells. Food Chem. Toxicol..

[3] Iordache S, Dunea D, Bøhler T, Iordache S, Dunea D, Bøhler T (2014). Current status of citizens protection against the risk of air pollution in urban areas. Methods to Assess the Effects of Air Pollution with Particulate Matter on Children’s Health (in Romanian).

[4] Mehmood K, Saifullah, Iqbal M, Rengel Z, Abrar MM (2020). Pakistan and India collaboration to improve regional air quality has never been more promising. Integr. Environ. Assess. Manag..

[5] Yang B, Guo J, Xiao C (2018). Effect of PM_2.5_ environmental pollution on rat lung. Environ. Sci. Pollut. Res..

[6] Baker KR, Foley KM (2011). A nonlinear regression model estimating single source concentrations of primary and secondarily formed PM2.5. Atmos. Environ..

[7] Goudarzi G, Alavi N, Geravandi S, Idani E, Behrooz HRA, Babaei AA, Alamdari FA, Dobaradaran S, Farhadi M, Mohammadi MJ (2018). Health risk assessment on human exposed to heavy metals in the ambient air PM 10 in Ahvaz, southwest Iran. Int. J. Biometeorol..

[8] Faraji Ghasemi F, Dobaradaran S, Saeedi R, Nabipour I, Nazmara S, Ranjbar Vakil Abadi D, Arfaeinia H, Ramavandi B, Spitz J, Mohammadi MJ, Keshtkar M (2020). Levels and ecological and health risk assessment of PM 2.5-bound heavy metals in the northern part of the Persian Gulf. Environ. Sci. Pollut. Res..

[9] Tahery N, Geravandi S, Goudarzi G, Shahriyari HA, Jalali S, Mohammadi MJ (2021). Estimation of PM 10 pollutant and its effect on total mortality (TM), hospitalizations due to cardiovascular diseases (HACD), and respiratory disease (HARD) outcome. Environ. Sci. Pollut. Res..

[10] Dastoorpoor M, Riahi A, Yazdaninejhad H, Borsi SH, Khanjani N, Khodadadi N, Khodadadi N, Mohammadi MJ, Aghababaeian H (2021). Exposure to particulate matter and carbon monoxide and cause-specific Cardiovascular-Respiratory disease mortality in Ahvaz. Toxin Rev..

[11] Moradi M, Mokhtari A, Mohammadi MJ, Hadei M, Vosoughi M (2022). Estimation of long-term and short-term health effects attributed to PM 2.5 standard pollutants in the air of Ardabil (using Air Q+ model). Environ. Sci. Pollut. Res..

[12] Shahriyari HA, Nikmanesh Y, Jalali S, Tahery N, Zhiani Fard A, Hatamzadeh N, Zarea K, Cheraghi M, Mohammadi MJ (2022). Air pollution and human health risks: mechanisms and clinical manifestations of cardiovascular and respiratory diseases. Toxin Rev..

[13] Mohammadi MJ, Fouladi Dehaghi B, Mansourimoghadam S, Sharhani A, Amini P, Ghanbari S (2022). Cardiovascular disease, mortality and exposure to particulate matter (PM): A systematic review and meta-analysis. Rev. Environ. Health.

[14] Borsi SH, Goudarzi G, Sarizadeh G, Dastoorpoor M, Geravandi S, Shahriyari HA, Mohammadi ZA, Mohammadi MJ (2022). Health endpoint of exposure to criteria air pollutants in ambient air of on a populated in Ahvaz City, Iran. Front. Public Health.

[15] Abbasi-Kangevari M, Malekpour MR, Masinaei M, Moghaddam SS, Ghamari SH, Abbasi-Kangevari Z, Rezaei N, Rezaei N, Mokdad AH, Naghavi M, Larijani B, Farzadfar F, Murray CJL (2023). Effect of air pollution on disease burden, mortality, and life expectancy in North Africa and the Middle East: A systematic analysis for the Global Burden of Disease Study 2019. Lancet Planet. Health.

[16] Nezhad ME, Goudarzi G, Babaei AA, Mohammadi MJ (2023). Characterization, ratio analysis, and carcinogenic risk assessment of polycyclic aromatic hydrocarbon compounds bounded PM10 in a southwest of Iran. Clin. Epidemiol. Glob. Health.

[17] El-Shahawy MA (2002). Prediction of air-pollution episodes. Bound. Layer Meteorol..

[18] Mehmood K, Bao Y, Cheng W, Khan MA, Siddique N, Abrar MM, Soban A, Fahad S, Naidu R (2022). Predicting the quality of air with machine learning approaches: Current research priorities and future perspectives. J. Clean. Prod..

[19] Boznar M, Lesjak M, Mlakar P (1993). A neural network-based method for short-term predictions of ambient SO_2_ concentrations in highly polluted industrial areas of complex terrain. Atmos. Environ. B Urban Atmos..

[20] Gardner MW, Dorling SR (1999). Neural network modelling and prediction of hourly NOx and NO_2_ concentrations in urban air in London. Atmos. Environ..

[21] Hadjiiski L, Hopke P (2000). Application of artificial neural networks to modeling and prediction of ambient ozone concentrations. J. Waste Manag. Assoc..

[22] Chaloulakou A, Grivas G, Spyrellis N (2003). Neural network and multiple regression models for PM10 prediction in Athens: A comparative assessment. J. Air Waste Manag. Assoc..

[23] Kolehmainen M, Martikainen H, Ruuskanen J (2001). Neural networks and periodic components used in air quality forecasting. Atmos. Environ..

[24] Nagendra SM, Khare M (2005). Modelling urban air quality using artificial neural network. Clean Technol. Environ. Policy.

[25] Chen L, Pai TY (2015). Comparisons of GM (1, 1), and BPNN for predicting hourly particulate matter in Dali area of Taichung City, Taiwan. Atmos. Pollut. Res..

[26] Bai Y, Li Y, Wang X, Xie J, Li C (2016). Air pollutants concentrations forecasting using back propagation neural network based on wavelet decomposition with meteorological conditions. Atmos. Pollut. Res..

[27] Wang D, Lu WZ (2006). Forecasting of ozone level in time series using MLP model with a novel hybrid training algorithm. Atmos. Environ..

[28] Durao RM, Mendes MT, Pereira MJ (2016). Forecasting O_3_ levels in industrial area surroundings up to 24 h in advance, combining classification trees and MLP models. Atmos. Pollut. Res..

[29] Lu WZ, Wang WJ, Wang XK, Yan SH, Lam JC (2004). Potential assessment of a neural network model with PCA/RBF approach for forecasting pollutant trends in Mong Kok urban air, Hong Kong. Environ. Res..

[30] Iliyas SA, Elshafei M, Habib MA, Adeniran AA (2013). RBF neural network inferential sensor for process emission monitoring. Control Eng. Pract..

[31] Shahraiyni HT, Sodoudi S, Kerschbaumer A, Cubasch U (2015). A new structure identification scheme for ANFIS and its application for the simulation of virtual air pollution monitoring stations in urban areas. Eng. Appl. Artif. Intell..

[32] Prasad K, Gorai AK, Goyal P (2016). Development of ANFIS models for air quality forecasting and input optimization for reducing the computational cost and time. Atmos. Environ..

[33] Maier HR, Dandy GC (2000). Neural networks for the prediction and forecasting of water resources variables: A review of modelling issues and applications. Environ. Model. Softw..

[34] Maier HR, Morgan N, Chow CW (2004). Use of artificial neural networks for predicting optimal alum doses and treated water quality parameters. Environ. Model. Softw..

[35] Pérez P, Trier A, Reyes J (2000). Prediction of PM_2.5_ concentrations several hours in advance using neural networks in Santiago, Chile. Atmos. Environ..

[36] McKendry IG (2002). Evaluation of artificial neural networks for fine particulate pollution (PM10 and PM2.5) forecasting. J. Air Waste Manag. Assoc..

[37] Chelani AB, Gajghate DG, Hasan MZ (2002). Prediction of ambient PM10 and toxic metals using artificial neural networks. J. Air Waste Manag. Assoc..

[38] Tecer LH (2007). Prediction of SO_2_ and PM concentrations in a coastal mining area (Zonguldak, Turkey) using an artificial neural network. Polish J. Environ. Stud..

[39] Pires JCM, Martins FG, Sousa SIV, Ferraz MCMA, Pereira MC (2008). Prediction of the daily mean PM10 concentrations using linear models. Am. J. Environ. Sci..

[40] Paschalidou AK, Karakitsios S, Kleanthous S, Kassomenos PA (2011). Forecasting hourly PM10 concentration in Cyprus through artificial neural networks and multiple regression models: Implications to local environmental management. Environ. Sci. Pollut. Res..

[41] Roy S, Adhikari GR, Renaldy TA, Jha AK (2011). Development of multiple regression and neural network models for assessment of blasting dust at a large surface coal mine. J. Environ. Sci. Technol..

[42] Kurt A, Oktay AB (2010). Forecasting air pollutant indicator levels with geographic models 3 days in advance using neural networks. Expert Syst. Appl..

[43] de Gennaro G, Trizio L, Di Gilio A, Pey J, Pérez N, Cusack M, Querol X (2013). Neural network model for the prediction of PM10 daily concentrations in two sites in the Western Mediterranean. Sci. Total Environ..

[44] Fu M, Wang W, Le Z, Khorram MS (2015). Prediction of particular matter concentrations by developed feed-forward neural network with rolling mechanism and gray model. Neural Comput. Appl..

[45] Fallahizadeh S, Kermani M, Esrafili A, Asadgol Z, Gholami M (2021). The effects of meteorological parameters on PM10: Health impacts assessment using AirQ+ model and prediction by an artificial neural network (ANN). Urban Clim..

[46] Bhardwaj R, Pruthi D (2020). Evolutionary techniques for optimizing air quality model. Procedia Comput. Sci..

[47] He J, Yu Y, Xie Y, Mao H, Wu L, Liu N, Zhao S (2016). Numerical model-based artificial neural network model and its application for quantifying impact factors of urban air quality. Water Air Soil Pollut..

[48] Maleki H, Sorooshian A, Goudarzi G, Baboli Z, Tahmasebi Birgani Y, Rahmati M (2019). Air pollution prediction by using an artificial neural network model. Clean Technol. Environ. Policy.

[49] Cakir S, Sita M (2020). Evaluating the performance of ANN in predicting the concentrations of ambient air pollutants in Nicosia. Atmos. Pollut. Res..

